# Virulence Potential of Fusogenic Orthoreoviruses

**DOI:** 10.3201/eid1806.111688

**Published:** 2012-06

**Authors:** Ann H. Wong, Peter K.C. Cheng, Mary Y.Y. Lai, Peter C.K. Leung, Kitty K.Y. Wong, W.Y. Lee, Wilina W.L. Lim

**Affiliations:** Centre for Health Protection, Hong Kong Special Administrative Region, People’s Republic of China

**Keywords:** fusogenic orthoreovirus, reassortment, antigenic characterization and phylogenetic analysis, virulence, viruses

## Abstract

Several severe respiratory virus infections that have emerged during the past decade originated in animals, including bats. In Indonesia, exposure to bats has been associated with increased risk of acquiring orthoreovirus infection. Although orthoreovirus infections are mild and self-limiting, we explored their potential for evolution into a more virulent form. We used conventional virus culture, electron microscopy, and molecular sequencing to isolate and identify orthoreoviruses from 3 patients in whom respiratory tract infection developed after travel to Indonesia. Virus characterization by plaque-reduction neutralization testing showed antigenic similarity, but sequencing of the small segment genes suggested virus reassortment, which could lead to increased virulence. Bats as a reservoir might contribute to virus evolution and genetic diversity, giving orthoreoviruses the potential to become more virulent. Evolution of this virus should be closely monitored so that prevention and control measures can be taken should it become more virulent.

## Abstract 2

Virus evolution should be monitored because frequent reassortment, which contributes to virus diversity, creates the potential for more severe infections.

The virus family *Reoviridae* comprises a diverse group of nonenveloped, segmented, double-stranded RNA viruses classified into 10 genera. Members of the genus *Orthoreovirus* are classified as fusogenic or nonfusogenic, depending on their ability to cause syncytium formation in cell culture. Genetically, orthoreoviruses contain 10 segments: 3 large, 3 medium, and 4 small (S1, S2, S3, and S4). The characteristic of the segmented genome facilitates virus reassortment, which contributes to virus diversity. Sequencing studies indicate that reassortment in nature is a major determinant of reovirus evolution (*1**,**2*).

In 2007, Chua et al. reported the isolation of Melaka virus, a novel fusogenic reovirus of bat origin, which has been associated with acute respiratory illness in humans (*3*). Subsequently, other related strains of bat-associated orthoreoviruses have been reported, including Kampar virus from Malaysia and Xi River virus from the People’s Republic of China (*4**,**5*). The role of bats as reservoirs of various zoonotic viruses, such as Nipah, Hendra, coronaviruses, and lyssavirus, has brought attention to the role of bats in virus evolution and emerging infections in humans (*6**–**10*).

We isolated and characterized 3 fusogenic orthoreoviruses from 3 travelers who had returned from Indonesia to Hong Kong during 2007–2010. Using a plaque-reduction neutralization test (PRNT), we confirmed infection in 2 of these patients and showed cross-reactivity of antibodies among the 3 virus strains. To track virus evolution and provide evidence of genetic reassortment, we used PCR sequencing and phylogenetic analysis to compare their genetic relatedness.

## Methods

### Case Descriptions

Patient 1 was a 51-year-old male tourist from Bali, Indonesia, who became ill in April 2007. This case has been reported elsewhere (*11*). The patient left Hong Kong before a serum sample could be obtained.

Patient 2 was a 26-year-old woman who sought care at a designated fever clinic during the outbreak of pandemic influenza in July 2009. She had a 2-day history of influenza-like illness (ILI), and oseltamivir was prescribed. Throat and nasal swab specimens were taken 3 days after onset of illness. Serum was collected 59 days after symptom onset. The patient recovered uneventfully. She reported having traveled to Bali, Indonesia, for 5 days and noticing ILI symptoms on the last day of her visit. While in Indonesia, she visited a safari park and entered bat caves on the second day. Her travel companions remained asymptomatic.

Patient 3 was a 29-year-old woman who sought care for ILI at the General Outpatient Clinic in Hong Kong in June 2010 after a recent visit to Indonesia. As part of sentinel surveillance for ILI, throat and nasal swab specimens were taken for routine isolation of respiratory viruses (*12*). Serum was collected 8 days after symptom onset.

### Virus Isolation

As part of routine surveillance to monitor influenza activity, throat and nasal swab specimens were inoculated into cells. The cell lines used for culture were MDCK, rhabdomyosarcoma, human laryngeal carcinoma, and rhesus monkey kidney (*12*).

### Virus Identification and Characterization

#### Immunofluorescence Testing

For samples that exhibited syncytial cytopathic effect (CPE) on cell culture, we used immunofluorescence testing with monoclonal antibodies to preliminarily identify viruses (*12*). We tested for viruses commonly associated with syncytial CPE formation on cell culture: respiratory syncytial virus, measles virus, and mumps virus. As part of routine investigation to exclude infection with influenza A(H1N1)pdm09 virus during the time of the pandemic influenza outbreak, a sample from patient 2 was also tested for influenza A.

#### Electron Microscopy

For viruses that had negative immunofluorescence results for respiratory syncytial, mumps, and measles virus monoclonal antibodies, we processed supernatant of tissue culture fluid for examination of virus morphologic appearance by electron microscopy. For negative staining, 1 drop of culture supernatant was adsorbed on Formvar carbon coated grid (1 min), stained with 3% phosphotungstic acid (pH 6.3) (1 min), and inactivated with ultraviolet irradiation before examination at 80 KV with a Philips transmission electron microscope (Eindhoven, the Netherlands).

#### PCR, Nucleotide Sequencing, and Phylogenetic Analysis

**Figure 1:**
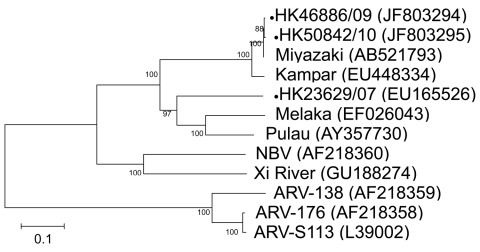
Phylogenetic tree of orthoreoviruses based on partial sequence alignment of the cell attachment protein (S1 gene segment). GenBank accession number for each sequence is in parentheses after the virus name. Numbers at nodes indicate bootstrap values based on 1,000 replicates. Dots indicate viruses isolated from 3 travelers who had returned from Indonesia to Hong Kong during 2007–2010. Scale bar indicates nucleotide substitutions per site. ARV, avian reovirus; NBV, Nelson Bay virus.

**Figure 2:**
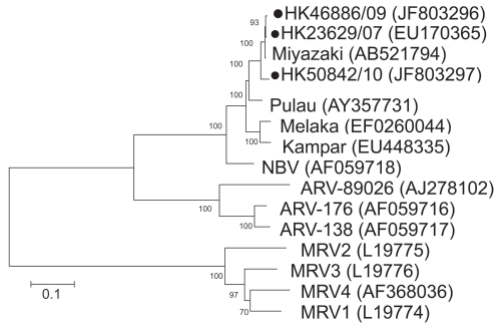
Phylogenetic tree of orthoreoviruses based on partial sequence alignment of the major inner capsid protein (S2 gene segment). GenBank accession number for each sequence is in parentheses after the virus name. Numbers at nodes indicate bootstrap values based on 1,000 replicates. Dots indicate viruses isolated from 3 travelers who had returned from Indonesia to Hong Kong during 2007–2010. Scale bar indicates nucleotide substitutions per site. ARV, avian reovirus; MRV, mammalian reovirus; NBV, Nelson Bay virus.

**Figure 3:**
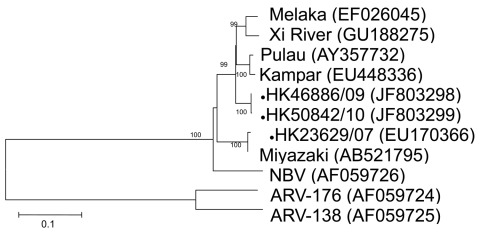
Phylogenetic tree of orthoreoviruses based on partial sequence alignment of the nonstructural protein (S3 gene segment). GenBank accession number for each sequence is in parentheses after the virus name. Numbers at nodes indicate bootstrap values based on 1,000 replicates. Dots indicate viruses isolated from 3 travelers who had returned from Indonesia to Hong Kong during 2007–2010. Scale bar indicates nucleotide substitutions per site. ARV, avian reovirus; NBV, Nelson Bay virus.

**Figure 4:**
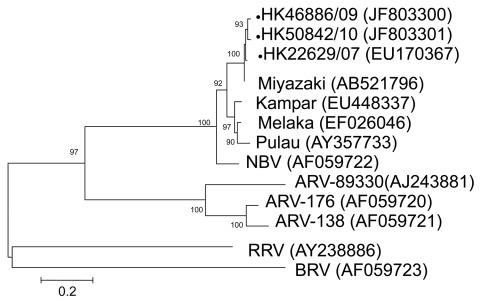
Phylogenetic tree of orthoreoviruses based on partial sequence alignment of the major outer capsid protein (S4 gene segment). GenBank accession number for each sequence is in parentheses after the virus name. Numbers at nodes indicate bootstrap values based on 1,000 replicates. Dots indicate viruses isolated from 3 travelers who had returned from Indonesia to Hong Kong during 2007–2010. Scale bar indicates nucleotide substitutions per site. ARV, avian reovirus; BRV, baboon reovirus; NBV, Nelson Bay virus; RRV, reptilian reovirus.

To further identify the virus and its phylogeny, we next designed primers based on published sequences of Melaka virus, selective for the S1 to S4 segments of orthoreovirus (*3*). In brief, by using a QIAamp Viral RNA Mini Kit (QIAGEN, Valencia, CA, USA) according to manufacturer’s instructions, we extracted viral RNA from 200 μL of culture supernatant of the 3 orthoreoviruses. Virus segments S1–S4 were amplified by using a QIAGEN One-Step RT-PCR Kit with primers shown in the Table. Reverse transcription PCR was performed as described (*3*). The amplified products were analyzed by 2% agarose gel electrophoresis. Sanger sequencing was conducted by using an ABI Prism 3130XL DNA Sequencer (Applied Biosystems, Foster City, CA, USA) and an ABI Prism Big Dye Terminator Cycle Sequencing Kit, version 3.1 (Applied Biosystems). Sequence alignment by using Simmonic and phylogenetic analysis of the partial sequences of segments S1 (1,501 nt), S2 (1,213 nt), S3 (1,078 nt), and S4 (1,113 nt) was performed by using MEGA4 software (www.megasoftware.net) with the neighbor-joining method. Sequences were submitted to GenBank and compared with other orthoreovirus sequences for genetic relatedness and evolution (Figure 1, Figure 2, Figure 3, Figure 4).

**Table:** Primers used to amplify the 4 small gene segments of fusogenic orthoreovirus from 3 travelers who had returned from Indonesia to Hong Kong during 2007–2010

Sequence, 5′ → 3′	Polarity
Segment 1	
ATGAGTGGTGATTGTGC	Forward
GATCTGATCGCGAAGCG	Reverse
GCTTTGATCATGACGATG	Forward
GTGTCGATTGAGAAAGTGA	Reverse
Segment 2	
CCCACGGACCAACGACAAC	Forward
AAAGCGCTTAGCTGAGAAGCG	Reverse
GAGACGTGGCCTAATATGTTG	Forward
GATGATTAGACCACGGCTGAG	Reverse
Segment 3	
GCGGGTACTGGGTCTCAGA	Forward
GCTTAGCACCAAAGGAAGTTACG	Reverse
GTCATCGCGAAGTTTTCAGAAC	Forward
CTCCTGTCGATGCTCACCA	Reverse
Segment 4	
CGCAAGCCCAGATGGAGGT	Forward
CGGAGTGACAATGACATGTTCAG	Reverse
CGTTTCCACATCATCGCTGTC	Forward
AGAGCATAGTGCGATGGTGTC	Reverse

#### PRNT

For PRNT, we first prepared a 1:5 diluted serum sample by using 2% minimal essential medium (MEM) and heat inactivation at 56°C for 30 min. We then performed serial double dilutions of serum samples from 1:5 to 1:2,560. Equal volumes of serum and virus mixture were incubated at 37°C for 2 h. Then 200 μL of serum and virus mixture with final dilutions of 1:10 to 1:5,120 was added to each of the 6-well plates containing a monolayer of confluent Vero cells (2 × 10^5^ cells/mL in 2% MEM). Serum, virus, and cell controls were included in the assay. After 1 h of virus absorption, the inoculum was discarded, plates were washed with 2% MEM, 4 mL of overlay LE agar was added, and the plates were incubated at 37°C in a CO_2_ incubator for 4 days. The end-point neutralizing antibody titer was determined by serum with the highest dilution that reduced plaques by 70% more than the virus control (*12*).

## Results

### Virus Identification

**Figure 5:**
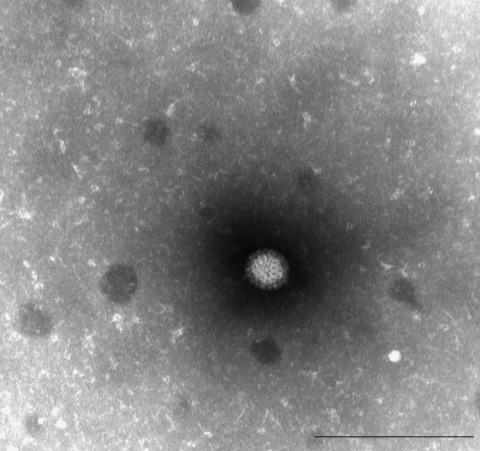
Electron micrograph of orthoreovirus HK50842/10. Scale bar = 200 nm.

All 3 orthoreoviruses (designated HK23629/07, HK46886/09, and HK50842/10) showed syncytial CPE in culture tubes after 3 to 4 days of incubation in each of the 4 cell lines. (Orthoreoviruses also cause CPE in MRC5 [human diploid lung] and Vero cells.) Immunofluorescent testing indicated that the samples were negative for influenza, respiratory syncytial, measles, and mumps viruses. Electron microscopy revealed virus morphologic appearance consistent with that of a reovirus (Figure 5).

### Neutralizing Antibody Titers

According to PRNT results, serum of patient 2 had a neutralizing antibody titer of 1,280 against HK46886/09 virus and HK23629/07 virus and a titer of 640 against HK50842/10 virus. Serum of patient 3 had a neutralizing antibody titer of 20 against all 3 viruses. The control serum had a neutralizing antibody titer <10.

### Nucleotide Sequences and Phylogeny

For the S1 virus cell attachment protein, the HK46886/09 and HK50842/10 viruses were clustered with the Miyazaki virus and related closely to the Kampar virus isolated in Malaysia; the S1 virus cell attachment protein of the HK23629/07 virus has been reported as similar but distinct from Melaka virus (*11*) (Figure 1). For the S2 major inner capsid protein and the S4 major outer capsid protein, all 3 viruses were clustered together and closely related to Miyazaki virus (Figure 2, Figure 4). For the S3 nonstructural protein, the HK46886/09 and HK50842/10 viruses were clustered together and similar to Kampar and Paula viruses, whereas the HK23629/07 virus was clustered with Miyazaki virus (Figure 3). Sequence analysis of the S1–S4 segments showed that HK46886/09 and HK50842/10 viruses were similar strains and different from the HK23629/07 virus. The difference in sequence homology between segments of the virus strains suggested reassortment events.

## Discussion

The isolation and epidemiologic studies of Melaka and Kampar virus in Malaysia provide evidence that a novel group of fusogenic orthoreovirus of bat origin is associated with human respiratory tract infection. The role of bats as reservoirs and sources of potential emerging viral infections has been extensively studied with regard to Nipah, Hendra, lyssavirus, and coronaviruses (*6**,**7**,**10*). Although we were unable to demonstrate rises in antibody titers because of a lack of paired acute-phase and convalescent-phase serum samples, we believe that the isolation of fusogenic orthoreoviruses, the presence of antibody to the viruses, and epidemiologic information suggest that orthoreoviruses were possibly associated with the patients’ upper respiratory tract infections.

Similar to infection caused by Melaka virus, the infection in the patients reported here was a mild upper respiratory infection followed by full recovery. Also, similar to the 7-day incubation period of the patient infected with Melaka virus, incubation periods for the patients reported here ranged from 4 to 7 days. The cross-reactivity of the 3 virus strains, demonstrated by PRNT, suggests that they were antigenically similar. Neutralizing antibodies appeared within 8 days of disease onset, and titers were still high at 2 months. Although all persons who had been in contact with the patients during their travel and at home were asymptomatic, no serum was available for further workup to determine subclinical infection or human-to-human transmission. Nevertheless, subclinical infection and transmission among close contacts have been demonstrated (*3**,**4*).

Phylogenetic analysis of the partial S1–S4 gene segments demonstrated genetic diversity of the viruses. Although the S2 major inner capsid proteins and S4 major outer capsid proteins of all 3 viruses were clustered together, they differed in the S1 virus cell attachment protein and S3 nonstructural protein. The S1 segments of HK46886/09 and HK50842/10 viruses were clustered together with the Miyazaki virus and related to the Kampar virus, but that of the HK23629/07 virus was similar to but distinct from the Melaka virus. The S3 segments of HK46886/09 and HK50842/10 viruses were clustered together and similar to those of the Kampar and Paula viruses, whereas the HK23629/07 virus shared similar homology with the Miyazaki virus. The difference in clustering based on the S1–S4 segments alone provides evidence for virus reassortment.

Epidemiologic information indicated that all 3 orthoreoviruses isolated were related to travel to Indonesia. Isolation of closely related orthoreoviruses within a short time in the region illustrates the genetic diversity of the virus and that reassortment events are not uncommon. The association of orthoreovirus infection with bat exposure showed that these abundant mammals are natural reservoirs and play a contributing role in virus evolution. In addition, the segmented nature of the virus genome also facilitates virus reassortment, which leads to virus diversity.

Outbreaks of emerging infections in the past decade, such as those caused by avian influenza (H5N1) virus, severe acute respiratory syndrome coronavirus, Nipah virus, and Hendra virus, have been associated with animal origins. Infections occurred in humans when the pathogens crossed the species barrier to expand their host range. Changes in human activities also bring humans in close contact with animals (*6**,**8**,**13**–**15*). Epidemiologic and molecular studies provide evidence that the pathogens isolated from humans shared a high degree of homology with viruses isolated from their animal reservoirs (*8**,**15*). The ability of a pathogen to emerge and cause outbreaks depends on the efficiency of its human-to-human spread. Recombination and reassortment events facilitate virus adaptation in the new host. Further study is warranted to unravel the possible virus spillover and adaptation of orthoreoviruses as a cause of human infection.

Although symptoms of orthoreovirus infection are mild and self-limiting, continued close monitoring of virus evolution and further study are essential. Changes in virulence would signal the need for prevention and control strategies.
